# Early combination of terlipressin and norepinephrine in the treatment of patients with septic shock

**DOI:** 10.1016/j.clinsp.2026.100988

**Published:** 2026-05-09

**Authors:** Zhenxing Ding, Mingqing Li, Yuanyuan Chen

**Affiliations:** Department of Emergency, the First Affiliated Hospital of Anhui Medical University, Hefei, Anhui, China

**Keywords:** Terlipressin, Norepinephrine, Septic shock, Outcome

## Abstract

**Objectives:**

This study set out to determine whether early use of Terlipressin (TP) as an adjunct to Norepinephrine (NE) could improve clinical outcomes in patients with septic shock.

**Material and Methods:**

In this prospective, randomized trial, 104 patients with septic shock were assigned to three groups receiving TP at different NE thresholds: Low-dose (L, ≥ 0.25 μg/kg/min), Mid-dose (M, ≥ 0.5 μg/kg/min), and High-dose (H, ≥ 0.75 μg/kg/min). Primary outcomes included 28-day mortality and vasopressor-free days. Secondary outcomes encompassed hemodynamic parameters, organ function recovery, inflammatory markers, and adverse events.

**Results:**

No significant difference was observed in 28-day mortality among groups (L: 42.86 %, M: 47.06 %, H: 51.43 %; *p* = 0.328). However, the L group exhibited significantly more vasopressor-free days (defined as days alive and free of all vasopressor support for ≥ 24 consecutive hours within the first 7-days after randomization; median 4.2, IQR 2.1–5.3) than the M (3.1-days) and H groups (2.3-days; *p* < 0.01). Key secondary outcomes showed superior hemodynamic stabilization in the L group, with shorter time to target MAP (4.2 vs. 5.6–6.5 h, *p* < 0.05) and accelerated lactate clearance (*p* < 0.01). Organ function recovery was significantly enhanced in the L group, demonstrated by reduced CRRT duration (3.5 vs. 6.8–7.5 days, *p* < 0.001), improved renal and hepatic function, higher platelet counts (215 vs. 130–165 × 10⁹/L, *p* < 0.001), and more rapid inflammatory resolution (Procalcitonin and CRP reduction, all *p* < 0.05). Adverse events were significantly lower in the L group (*p* < 0.001).

**Conclusions:**

Early low-dose TP adjunctive therapy reduces NE dependence, enhances hemodynamic stability, promotes multi-organ function recovery, and improves safety profile in septic shock, without significantly affecting 28-day mortality. These findings support its role as a beneficial adjunct vasopressor when initiated at lower NE doses.

## Highlights

• Early low-dose terlipressin reduced norepinephrine dependence in septic shock.
• Low-dose terlipressin increased vasopressor-free days within the first 7-days.
• Earlier terlipressin improved hemodynamic stabilization and lactate clearance.
• Low-dose terlipressin promoted faster renal, hepatic, and platelet recovery.
• Early low-dose terlipressin was associated with fewer ischemic adverse events.

## Introduction

Sepsis is defined as life-threatening organ dysfunction resulting from a dysregulated host response to infection.^1^ Septic shock, a severe subset of sepsis, is characterized by profound circulatory and cellular/metabolic abnormalities, which is clinically identified by persistent hypotension ‒ specifically, a Mean Arterial Pressure (MAP) ≤ 65 mmHg and hyperlactatemia (lactate ≥2 mmoL/L) despite adequate fluid resuscitation.^2^ This condition is associated with significantly elevated mortality due to progressive dysfunction in perfusion and cellular metabolism.^3^ Current management guidelines emphasize early hemodynamic stabilization through prompt fluid resuscitation, vasopressor support, respiratory assistance, and timely administration of antibiotics.^4^ Achieving and maintaining stable hemodynamics is critical for improving outcomes, with evidence indicating that rapid and adequate fluid resuscitation and early administration of vasoactive agents significantly reduce mortality in patients with septic shock.^5^

Norepinephrine (NE) is recommended as the first-line vasoactive agent in current guidelines for the management of septic shock to correct hypotension.^6^, ^7^, ^8^ However, high-dose NE administration is associated with an increased risk of fatal arrhythmias in critically ill patients,^6^ and prolonged use may lead to catecholamine resistance.^7^ Accordingly, guidelines suggest that adjunctive vasopressor therapy ‒ such as vasopressin ‒ should be considered when NE dosage reaches approximately 0.5 μg/(kg/min), in order to achieve the target MAP earlier and reduce NE requirements.^3^ Although vasopressin has been shown to reduce both NE dosage and the incidence of arrhythmias, it has not demonstrated a consistent mortality benefit in patients with septic shock.^8^, ^9^, ^10^, ^11^

Notably, despite Terlipressin (TP) ‒ a synthetic long-acting vasopressin analog ‒ showing potential as an alternative adjunct for septic shock, critical knowledge gaps remain regarding its optimal timing and dosing.^12^ A key mechanistic distinction between TP and vasopressin further lies in TP’s higher selectivity for the V1 receptor, a property that may minimize off-target effects while enhancing targeted vasoconstriction. Previous clinical studies, however, have not consistently demonstrated clear clinical benefits for TP, which may be attributable to variations in dosing strategies and timing of initiation.^13^^,^^14^ Nevertheless, emerging evidence indicates that TP may offer advantages in specific contexts, such as reducing NE dependency, improving creatinine clearance, and stabilizing hemodynamics.^15^ Some studies also suggest that continuous infusion of TP could be more effective than vasopressin in restoring hemodynamic stability and may be associated with fewer adverse events.^14^^,^^16^ These findings highlight the potential value of optimized dosing and timing in the use of TP as an adjunctive therapy in septic shock.

The primary objective of this study was to determine how early combined use of TP and NE affects clinical outcomes in patients with septic shock, and to further explore the optimal timing and dosing of TP for this therapy.

## Material and methods

### Inclusion and exclusion criteria

Patients were eligible if they met all of the following criteria: 1) Diagnosis of septic shock according to the Sepsis-3 International Consensus Definitions,^3^ 2) Requirement for vasopressor support to maintain MAP ≥ 65 mmHg following adequate fluid resuscitation, defined as administration of at least 30 mL/kg of crystalloid solution within the first 3 h, and 3) Serum lactate level ≥ 2 mmoL/L.

Exclusion criteria: 1) Age < 18-years, 2) Evidence of left ventricular dysfunction, 3) Acute coronary syndrome or recent acute myocardial infarction, 4) Significant valvular heart disease, 5) Suspected or confirmed mesenteric ischemia or vasospastic disorders, 6) Chronic renal failure (baseline eGFR < 15 mL/min/1.73 m^2^ or requiring chronic dialysis), 7) Pregnancy or lactation, 8) History of solid organ transplantation, 9) Raynaud’s phenomenon or other severe peripheral vascular diseases, 10) Known hypersensitivity to TP or related vasoactive agents, or 11) Prior administration of TP during the current hospitalization.

Exclusion determinations were made through a comprehensive review of all available medical records, structured interviews with patients and/or family members at admission, and results from diagnostic evaluations conducted during hospitalization, including echocardiography, thoracic/abdominal computed tomography, ultrasonography, and laboratory assessments.

### Study design

This prospective, randomized, single-blind interventional trial enrolled patients with septic shock admitted to the adult and emergency intensive care units of the First Affiliated Hospital of Anhui Medical University between February 2021 and February 2023. The study protocol was reviewed and approved by the Institutional Ethics Committee of the First Affiliated Hospital of Anhui Medical University (Approval n°NKZ060) before the initiation of patient enrollment, ensuring full ethical compliance. All participants or their legal surrogates provided written informed consent prior to inclusion. The trial was subsequently registered with the Chinese Clinical Trial Registry (ChiCTR2400082897, https://www.chictr.org.cn/showproj.html?proj=219829) due to an administrative delay in online submission. Although the formal registry entry was completed after the completion of patient recruitment, this does not affect the study’s prospective design, ethical validity, or adherence to institutional and national regulatory standards.

Randomization was implemented using a simple randomization method. The random allocation sequence was generated via the “random number generator” function in GraphPad Prism 9 software (GraphPad Software, San Diego, CA, USA), ensuring each patient had an equal probability of being assigned to any of the three groups. To prevent selection bias, allocation concealment was achieved using sealed opaque envelopes. Specifically, an independent statistician who was not involved in patient enrollment or outcome assessment filled numbered, sealed opaque envelopes with the allocation results (L group/M group/H group) according to the random sequence. When an eligible patient completed informed consent and was confirmed for enrollment, the enrolling investigator opened the sealed envelope corresponding to the patient's enrollment order to determine the treatment group, and then initiated the intervention protocol.

Patients were randomly assigned via computer-generated numbers to one of three treatment groups: Low-dose (L), Medium-dose (M), or High-dose (H). Researchers were aware of treatment assignments, while patients were blinded to the intervention.

### Sample-size estimation

Sample-size estimation was performed a priori using G*Power 3.1 software (Heinrich-Heine-Universität Düsseldorf, Germany), based on the primary outcomes (28-day mortality and vasopressor-free days) and evidence from high-quality, retrievable meta-analyses on Terlipressin (TP) in septic shock:

For 28-day mortality: A systematic review and meta-analysis^14^ evaluating TP’s effects on septic shock reported that adjunctive TP was associated with a trend toward reduced mortality, with observed mortality rates ranging from 42 % to 52 % in TP-treated subgroups. The authors hypothesized a clinically meaningful 12 % absolute reduction in mortality (from 55 % in the High-dose [H] group to 43 % in the Low-dose [L] group) ‒ a threshold consistent with the mortality variability reported in this meta-analysis. With α = 0.05 (two-sided) and power (1−β) = 0.80, the calculated sample size per group was 32 patients.

For vasopressor-free days: A randomized controlled pilot study^16^ comparing continuous TP vs. vasopressin in septic shock observed a median difference of 1.5-days in vasopressor-free days between groups. The authors assumed a 2-day median difference (4.2-days in L group vs. 2.2-days in H group) ‒ a clinically relevant threshold for reducing catecholamine exposure. With α = 0.05, power = 0.80, and an estimated standard deviation of 2.3-days (derived from this pilot study), 30 patients per group were required.

To address potential dropouts (e.g., consent withdrawal, early death within 48-hours), the authors inflated the sample size by 15 %, resulting in a target of 35 patients per group (total 105). After excluding 1 patient who withdrew consent post-randomization, the final 104 participants remained consistent with the a priori estimate ‒ ensuring sufficient power to detect the predefined clinically meaningful differences for both primary outcomes.

### Intervention protocol

All patients received NE starting at 0.1 μg/(kg/min). TP was initiated as an adjunctive vasopressor when NE reached the following pre-specified thresholds without achieving a target MAP ≥ 65 mmHg:

• L group: NE ≥ 0.25 μg/(kg·min)
• M group: NE ≥ 0.5 μg/(kg·min)
• H group: NE ≥ 0.75 μg/(kg·min)

TP was administered as a continuous intravenous infusion at 0.01–0.02 μg/(kg/min). If MAP remained below target despite maximum TP dosing, NE was up-titrated accordingly. Study drugs were prepared in 0.9 % sodium chloride solution (NE 20 mg/50 mL; TP 2 mg/50 mL). Additional supportive care included dobutamine for cardiac index ≤ 3.0 L/min/m^2^, red blood cell transfusion for hemoglobin < 7 g/dL, and adherence to international sepsis management guidelines. Vasoactive agents were weaned once MAP was maintained ≥ 65 mmHg for 12-hours or if MAP exceeded 80 mmHg. TP was tapered before NE in all groups.

### Data collection

Data were manually extracted from electronic medical records and included demographics, vital signs, SOFA (Sequential Organ Failure Assessment) (at enrollment and day-7) and APACHE II (Acute Physiology and Chronic Health Evaluation II) scores (at enrollment), laboratory results (hematologic, biochemical, and coagulation parameters), duration of mechanical ventilation, renal replacement therapy, vasopressor dosing, infection sources, organ function and adverse events.

### Outcomes

The coprimary outcomes were 28-day all-cause mortality and vasopressor-free days through day-7 (defined as the number of days alive and free of vasopressor support for at least 24 consecutive hours within the first 7-days after randomization).

Key secondary outcomes included hemodynamic parameters (time to achieve MAP ≥ 65 mmHg, NE requirement reduction at 48 h after TP initiation, n [ %]), change in SOFA score from baseline to day-7, and the incidence of serious adverse events, including ischemic complications.

Additional secondary endpoints comprised duration of mechanical ventilation, requirement and duration of renal replacement therapy, and organ function.

### Statistical analysis

Continuous variables are presented as mean ± Standard Deviation (SD) if normally distributed, or as median with Interquartile Range (IQR) if non-normally distributed. The Shapiro-Wilk test was used to verify the distribution of continuous variables before selecting statistical tests: variables with a Shapiro-Wilk p-value > 0.05 were considered normally distributed and analyzed using parametric methods, while those with *p* < 0.05 were treated as non-normally distributed and analyzed with non-parametric methods. Categorical variables are reported as frequencies and percentages. For comparisons among the three groups, one-way analysis of variance (ANOVA) with post hoc Tukey testing was used for normally distributed continuous variables, while the Kruskal-Wallis test followed by Dunn’s multiple comparison adjustment was applied for non-normally distributed variables. Categorical variables were compared using the chi-square test or Fisher’s exact test, as appropriate. Regarding missing data, all enrolled patients had complete data for primary outcomes (28-day mortality, vasopressor-free days). For secondary endpoints, missing data accounted for < 2 % of total observations and were handled using the “Last Observation Carried Forward” (LOCF) method, which is consistent with common practices in septic shock clinical trials to maintain data integrity without introducing excessive bias. All analyses were performed using GraphPad Prism 9 (GraphPad Software, San Diego, CA, USA), with a two-sided p-value <0.05 considered statistically significant.

## Results

### Patient characteristics and study flow

**Fig. 1:**
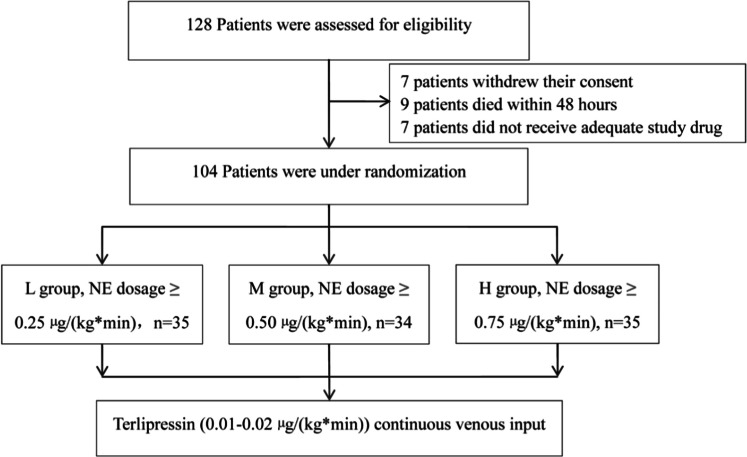
Flow chart.

A total of 128 patients with septic shock were randomized between February 2021 and February 2023. After exclusions ‒ 7 who withdrew consent, 7 who did not require prolonged study drug infusion due to rapid clinical improvement, and 9 who died within 48-hours (reason: < 12-hour TP exposure insufficient for core secondary endpoints requiring 24–48 h of follow-up, and deaths attributed to severe baseline illness and irreversible septic shock) ‒ 104 patients were included in the final intention-to-treat analysis (Fig. 1).

The patients were allocated to three groups: low-dose (L group, *n* = 35; mean age 59.6 ± 17.0 years), medium-dose (M group, *n* = 34; 59.3 ± 13.9 years), and high-dose (H group, *n* = 35; 61.3 ± 15.5 years). Baseline demographic and clinical characteristics were well-balanced across all groups, with no statistically significant differences observed in sex distribution, age, disease severity scores (APACHEII, SOFA), past medical history, site of infection, and hemodynamic parameters (Table 1).

**Table 1:** Clinical characteristics from enrolled patients.

Parameters	L Group (*n* = 35)	M Group (*n* = 34)	H Group (*n* = 35)	p
Male sex, n (%)	20 (57.1)	19 (55.9)	21 (60.0)	0.943
Age (years)	59.6 ± 17.0	59.3 ± 13.9	61.3 ± 15.5	0.872
APACHEII score	28.5 ± 5.5	27.8 ± 5.2	29.1 ± 5.8	0.654
SOFA score	13.5 ± 2.5	13.2 ± 2.8	13.8 ± 2.6	0.732
Past Medical History, n (%)				
Hypertension	18 (51.4)	16 (47.1)	17 (48.6)	0.924
Diabetes mellitus	12 (34.3)	11 (32.4)	13 (37.1)	0.901
Chronic lung disease	7 (20.0)	6 (17.6)	8 (22.9)	0.853
Heart failure	5 (14.3)	6 (17.6)	7 (20.0)	0.812
Atrial fibrillation	6 (17.1)	5 (14.7)	7 (20.0)	0.831
Cancer	7 (20.0)	8 (23.5)	9 (25.7)	0.842
Site of Infection, n (%)				
Pulmonary	14 (40.0)	13 (38.2)	15 (42.9)	0.923
Intra-abdominal	8 (22.9)	7 (20.6)	9 (25.7)	0.865
Genitourinary	7 (20.0)	8 (23.5)	6 (17.1)	0.782
Bloodstream	6 (17.1)	5 (14.7)	7 (20.0)	0.831
Skin/Soft tissue	3 (8.6)	4 (11.8)	2 (5.7)	0.654
Other/Unknown	4 (11.4)	3 (8.8)	5 (14.3)	0.772
Hemodynamic Parameters				
Baseline MAP, mmHg, mean ± SD	54.2 ± 6.8	53.8 ± 7.2	52.9 ± 6.5	0.721
Initial lactate, mmol/L, mean ± SD	5.8 ± 1.9	5.9 ± 2.1	6.1 ± 2.3	0.892

SOFA, Sequential Organ Failure Assessment; APACHEII, Acute Physiology and Chronic Health Evaluation II; SD, Standard Deviation.

### Primary outcomes

No significant difference in 28-day survival was observed among the three groups (*p* = 0.328) (Table 2). The 28-day all-cause mortality rates were 42.86 % (15/35) in the L group, 47.06 % (16/34) in the M group, and 51.43 % (18/35) in the H group. Notably, while these RRs suggest a directional trend toward reduced mortality in the L group, the wide 95 % CIs (crossing 1.0) and non-significant p-value (*p* = 0.328) indicate that this trend lacks statistical robustness and should not be over-interpreted as evidence of a survival benefit. However, vasopressor-free days within the first 7-days were significantly higher in the L group (median 4.2-days, IQR 2.1–5.3) compared to the M group (median 3.1-days, IQR 1.2–4.1) and H group (median 2.3-days, IQR 1.1–3.2; *p* < 0.01) (Table 2).

**Table 2:** Comparison of primary and secondary outcomes among the three groups.

Outcome Measure	Low-dose Group (L) (*n* = 35)	Medium-dose Group (M) (*n* = 34)	High-dose Group (H) (*n* = 35)	p-value
Primary Outcomes				
28-day all-cause mortality, n (%)	15 (42.86)	16 (47.06)	18 (51.43)	0.328
Vasopressor-free days, days, median (IQR)	4.2 (2.1–5.3)	3.1 (1.2–4.1)	2.3 (1.1–3.2)	<0.01
Secondary Outcomes				
Hemodynamic Parameters				
Time to achieve MAP ≥65 mmHg, hours, mean ± SD	4.2 ± 1.1	5.6 ± 2.3	6.5 ± 2.8	<0.05
Norepinephrine requirement reduction, n (%)	30 (85.7)	25 (73.5)	22 (62.9)	0.021
Lactate clearance at 72-hours, (%), mean ± SD	68.4 ± 12.3	52.1 ± 15.6	45.3 ± 14.8	< 0.01
Organ Function				
ΔSOFA score (Baseline to day-7), mean ± SD	−8.4 ± 2.5	−5.9 ± 2.8	−5.3 ± 2.6	<0.01
Duration of CRRT, days, mean ± SD	3.5 ± 1.2	6.8 ± 2.4	7.5 ± 2.6	<0.001
Duration of mechanical ventilation, days, mean ± SD	5.2 ± 2.1	7.8 ± 2.9	8.5 ± 3.2	0.432

IQR, Interquartile Range; MAP, Mean Arterial Pressure; SOFA, Sequential Organ Failure Assessment; CRRT, Continuous Renal Replacement Therapy.

Data are presented as n (%), mean ± Standard Deviation (SD), or median (IQR) as appropriate.

p-values represent between-group comparisons using log-rank test for survival data, Kruskal-Wallis test for non-normally distributed continuous data, and one-way ANOVA for normally distributed continuous data.

Vasopressor-free days defined as the number of days within the first 7-days after randomization during which the patient did not receive any vasopressor support for at least 24 consecutive hours.

### Secondary outcomes

The addition of TP was associated with improved hemodynamic stability, as indicated by a reduced NE requirement and a shorter time to achieve target mean arterial pressure (MAP ≥ 65 mmHg; L group: 4.2 ± 1.1 h, M group: 5.6 ± 2.3 h, H group: 6.5 ± 2.8 h; *p* < 0.05) (Table 2). Serum lactate levels decreased significantly in all groups over 72-hours, with the most pronounced reduction observed in the L group (*p* < 0.01) (Table 2).

Marked improvements in organ function were observed in the L group compared to the other groups. The mean ΔSOFA score (from baseline to day-7) was significantly greater in the L group (−8.4 ± 2.5) compared to the M (−5.9 ± 2.8) and H groups (−5.3 ± 2.6; *p* < 0.01), indicating a more substantial improvement in organ dysfunction. Similarly, the mean duration of CRRT was significantly shorter in the L group (3.5 ± 1.2 days) compared to the M (6.8 ± 2.4 days) and H groups (7.5 ± 2.6 days; *p* < 0.001). No significant differences were observed in the duration of mechanical ventilation among the groups (*p* = 0.432).

### Among-group comparison of laboratory values

Correspondingly, serum creatinine levels decreased more rapidly in the L group, with significantly lower values at day-7 (89.3 ± 22.4 μmol/L) compared to the M (120.6 ± 35.2 μmol/L) and H groups (158.9 ± 40.8 μmol/L; *p* < 0.001). Hepatic function also showed greater improvement in the L group, as evidenced by a more substantial reduction in total bilirubin levels by day-7 (22.1 ± 7.5 μmol/L) versus the M (30.5 ± 9.2 μmol/L) and H groups (39.8 ± 12.1 μmol/L; *p* = 0.015) (Table 3).

**Table 3:** Comparison of laboratory parameters between the three group.

Parameter	Group	Baseline	Day 3	Day 7	Day 28	p-value (Between-group, Δ D7)
Creatinine (μmol/L)	L	210.5 ± 45.2	125.8 ± 30.1*†	89.3 ± 22.4*†	78.5 ± 20.1	0.003
	M	205.8 ± 42.3	165.4 ± 38.7*	120.6 ± 35.2*	95.3 ± 28.4	
	H	215.2 ± 50.1	195.7 ± 42.5	158.9 ± 40.8*	110.7 ± 35.6	
Total Bilirubin (μmol/L)	L	45.2 ± 12.8	38.5 ± 10.2	22.1 ± 7.5*†	16.8 ± 6.2	0.015
	M	43.7 ± 11.5	40.1 ± 11.8	30.5 ± 9.2*	22.5 ± 7.8	
	H	47.1 ± 14.3	45.8 ± 13.6	39.8 ± 12.1	28.9 ± 9.5	
Platelet count (× 10⁹/L)	L	85 ± 25	105 ± 30	215 ± 45*†	245 ± 50	<0.001
	M	82 ± 23	90 ± 28	165 ± 40*	210 ± 45	
	H	88 ± 27	84 ± 25	130 ± 35*	185 ± 40	
Procalcitonin (μg/L)	L	35.2 [18.5‒52.1]	5.1 [2.2‒10.3]*†	0.8 [0.3‒2.1]*†	0.2 [0.1‒0.5]	0.008
	M	33.8 [16.8‒50.2]	8.5 [3.8‒15.7]*	1.5 [0.5‒3.5]*	0.3 [0.1‒0.6]	
	H	36.5 [19.1‒55.0]	12.8 [5.2‒20.1]*	2.8 [1.0‒5.2]*	0.5 [0.2‒1.0]	
CRP (mg/L)	L	185 ± 45	95 ± 30*†	35 ± 15*†	12 ± 8	0.01
	M	180 ± 42	110 ± 35*	50 ± 20*	18 ± 10	
	H	190 ± 48	125 ± 40*	65 ± 25*	25 ± 12	

Hematological recovery was enhanced in the L group, with platelet counts increasing to 215 ± 45 × 10^9^/L by day-7, significantly higher than in the M (165 ± 40 × 10^9^/L) and H groups (130 ± 35 × 10^9^/L; *p* < 0.001) (Table 3).

Inflammatory resolution was accelerated in the L group. Procalcitonin (PCT) levels declined most rapidly in the L group (0.8 [0.3‒2.1] μg/L by day-7), followed by the M (1.5 [0.5‒3.5] μg/L) and H groups (2.8 [1.0–5.2] μg/L; *p* = 0.008). Similarly, C-Reactive Protein (CRP) levels decreased to 35±15 mg/L in the L group by day-7, significantly lower than in the M (50 ± 20 mg/L) and H groups (65 ± 25 mg/L; *p* = 0.01) (Table 3).

### Adverse events

The incidence of serious adverse events was significantly lower in the L group than in the M and H groups (*p* < 0.001) (Table 4). This difference was primarily driven by a reduced incidence of digital ischemia and acute renal failure in the L group.

**Table 4:** Comparison of serious adverse events among the three groups.

Serious Adverse Event	L Group (*n* = 35)	M Group (*n* = 34)	H Group (*n* = 35)	p-value
Any SAE, n (%)	4 (11.4)	10 (29.4)	13 (37.1)	<0.001
Acute renal failure, n (%)	2 (5.7)	5 (14.7)	7 (20.0)	0.15
Coagulation dysfunction, n (%)	1 (2.9)	3 (8.8)	4 (11.4)	0.35
Arrhythmia, n (%)	2 (5.7)	4 (11.8)	5 (14.3)	0.42
Digital ischemia, n (%)	0 (0.0)	3 (8.8)	6 (17.1)	0.02

SAE, Serious Adverse Event.

Comparisons for individual events performed using Fisher's exact test, Data are presented as n (%).

## Discussion

Despite advances in critical care, mortality due to septic shock remains unacceptably high, ranging from 41.9 %‒50.9 %^17^^,^^18^ to over 80 % in severe cases.^19^ Excessive reliance on high-dose NE ‒ particularly beyond 1.0 μg/(kg/min) ‒ is associated with markedly increased mortality and adverse effects.^20^ While early NE administration improves outcomes,^21^ catecholamine resistance and dose-related toxicity often develop, necessitating adjunctive strategies. This study evaluated the early combination of low-dose TP with NE, an approach grounded in the pathophysiological rationale of mitigating catecholamine overload while providing sustained vasopressor support through selective V₁ receptor activation.^22^, ^23^, ^24^, ^25^

These findings demonstrate that early low-dose TP supplementation significantly reduced NE requirements and increased vasopressor-free days within the first week, consistent with previous reports.^26^, ^27^, ^28^, ^29^ Although 28-day mortality did not differ significantly across groups, the L group showed notable improvements in multiple secondary outcomes. More notably, TP administration was associated with accelerated lactate clearance and earlier hemodynamic stabilization, observations consistent with potential improvements in tissue perfusion and potentially linked to enhanced microcirculatory function ‒ a finding supported by experimental models.^30^^,^^31^

The organ-protective effects observed in the present study are particularly noteworthy. Patients receiving early low-dose TP exhibited significantly shorter duration of renal replacement therapy and more rapid improvement in renal function, aligning with TP's proposed reno-protective effects via V₁-mediated restoration of renal perfusion pressure and reduction in catecholamine-induced vasoconstriction.^29^^,^^31^ Hepatic function also showed greater improvement, with significantly lower bilirubin levels in the L group. Additionally, enhanced hematological recovery was observed, as indicated by significantly higher platelet counts in the L group compared to both M and H groups.

The anti-inflammatory effects of early low-dose TP represent another important hypothesis-generating finding. PCT and CRP levels declined most rapidly in the L group, suggesting that early intervention with appropriate dosing may modulate the excessive inflammatory response characteristic of septic shock.^32^

Importantly, the L group experienced markedly fewer serious adverse events ‒ particularly digital ischemia and acute renal failure ‒ compared to both the M and H groups. This safety profile underscores the therapeutic window of TP and suggests that lower dosing mitigates the risk of non-selective receptor activation and ischemic complications.^8^, ^9^, ^10^, ^11^^,^^24^ The present results are consistent with earlier studies indicating that a TP infusion rate of 0.021 µg/(kg/min) is both safe and effective.^16^^,^^33^, ^34^, ^35^

### Limitations of the study

Several limitations should be acknowledged. First, the single-center design and moderate sample size may limit generalizability. Notably, this study was conducted in a Chinese cohort, and potential differences in baseline characteristics or sepsis etiologies between this population and Western cohorts could further impact the external validity of the present findings regarding Terlipressin (TP) response. This cohort had lower rates of chronic obstructive pulmonary disease and coronary artery disease ‒ which may explain low-dose TP’s favorable safety (0 % digital ischemia in L group; Table 4) ‒ and fewer device-related bloodstream infections, potentially enhancing TP’s NE-sparing effect, given gram-negative sepsis (dominant in the cohort) is more responsive to TP’s vasoconstriction.

Second, although group allocation was randomized, the single-blind design (researchers aware of treatment assignments, patients blinded) introduced potential performance bias ‒ most notably in NE titration. Given clinicians knew the pre-specified NE thresholds for TP initiation (≥ 0.25 μg/kg/min for L group, ≥ 0.5 μg/kg/min for M group, ≥ 0.75 μg/kg/min for H group), they might have adjusted NE dosing differentially: for L group patients, clinicians could have been more conservative with NE up-titration to initiate TP earlier; for H group patients, they might have tolerated higher NE levels longer. This could have confounded outcomes like vasopressor-free days and NE requirement reduction. Unblinded assessment might also have introduced subtle detection bias for the secondary outcome. Future multi-center trials should adopt double-blind designs to mitigate these biases and refine TP dosing protocols.

## Conclusions

In this randomized trial, early low-dose TP adjunctive therapy reduced NE requirements, increased vasopressor-free days, and promoted multi-organ recovery in septic shock. Although 28-day mortality remained unchanged, the improvements in key secondary outcomes and the favorable safety profile support its potential role as an adjunct vasopressor. These results emphasize the value of dose optimization and early intervention in septic shock management. Further studies are needed to define the optimal target population and timing for TP therapy.

## Patient permission/consent declarations

The committee on medical ethics of the First Affiliated Hospital of Anhui Medical University approved this study (n°NKZ060), and the informed consents in this study were signed by all patients.

## Data availability

The data that support the findings of this study are available from the corresponding author upon reasonable request.

## Funding

This work was funded by the 10.13039/501100018628Department of Education of Anhui Province (2025AHGXZK30415), and 10.13039/501100002947Anhui Medical University (n°2023xkj131).

## Declaration of competing interest

The authors declare no conflicts of interest.
